# Systemic Delivery of Oncolytic Viruses: Hopes and Hurdles

**DOI:** 10.1155/2012/805629

**Published:** 2012-01-31

**Authors:** Mark S. Ferguson, Nicholas R. Lemoine, Yaohe Wang

**Affiliations:** ^1^Centre for Molecular Oncology, Barts Cancer Institute, Queen Mary University of London, London EC1M 6BQ, UK; ^2^Sino-British Research Centre for Molecular Oncology, Zhengzhou University, Zhengzhou 450052, China

## Abstract

Despite recent advances in both surgery and chemoradiotherapy, mortality rates for advanced cancer remain high. There is a pressing need for novel therapeutic strategies; one option is systemic oncolytic viral therapy. Intravenous administration affords the opportunity to treat both the primary tumour and any metastatic deposits simultaneously. Data from clinical trials have shown that oncolytic viruses can be systemically delivered safely with limited toxicity but the results are equivocal in terms of efficacy, particularly when delivered with adjuvant chemotherapy. A key reason for this is the rapid clearance of the viruses from the circulation before they reach their targets. This phenomenon is mainly mediated through neutralising antibodies, complement activation, antiviral cytokines, and tissue-resident macrophages, as well as nonspecific uptake by other tissues such as the lung, liver and spleen, and suboptimal viral escape from the vascular compartment. A range of methods have been reported in the literature, which are designed to overcome these hurdles in preclinical models. In this paper, the potential advantages of, and obstacles to, successful systemic delivery of oncolytic viruses are discussed. The next stage of development will be the commencement of clinical trials combining these novel approaches for overcoming the barriers with systemically delivered oncolytic viruses.

## 1. Introduction

Cancer remains a major health problem and is the 5th leading cause of death worldwide [1]. There have been many advances in the last few decades both in surgical care and chemoradiotherapy regimes. Certainly this has contributed to improved survival rates for commonly occurring cancers. However, relapse and disease progression are still all too common occurrences in modern medical practice. A variety of novel adjuvant therapies have been developed over the last decade, and oncolytic viruses have been particularly promising members of this cohort.

Oncolytic viruses came to medical prominence in the 19th century when coincidental viral infections were observed to cause regression of some forms of haematological malignancies. Rabies inoculation was also demonstrated to regress a patient's advanced cervical carcinoma [2]. A succession of studies in the 1950s and 60s were unable to establish oncolytic viral therapy as a viable anti-cancer modality. As a result, the field remained a medical curiosity until the advent of genetic engineering in the late 1980s. In the last decade, there have been rapid advancements in the oncolytic viral therapy field. Naturally occurring oncolytic viruses have been identified such as Vaccinia virus, Reovirus, and Newcastle disease virus. These viruses naturally preferentially infect tumour cells whilst sparing normal tissue. However, other viruses have been identified that once attenuated are also successful oncolytic agents such as herpes simplex virus type 1 and Adenovirus. These viruses have then been engineered to be more tumour specific and less pathogenic to normal tissues [2]. This has been achieved by a variety of modifications [3]. Herpes simplex virus has had two of its latency genes deleted (ICP0 & ICP4) and only has one copy of its virulence factor, *γ*134.5, remaining. As a further level of safety its thymidine kinase gene has been deleted. Deletion of the thymidine kinase gene means that viruses can only replicate efficiently in cells with upregulation of the EGFR/Ras signalling pathway, which is commonly the case in tumour cells [4, 5]. This approach has been widely employed successfully with Vaccinia virus developed for clinical trial use. Adenoviruses used in clinical trials have E1B 55 K gene deleted, which is involved in late viral RNA export and restricts E1B 55 K-deleted adenovirus replication in normal primary cells [6]. All of these modifications are designed to make the oncolytic viruses more tumour specific since these gene deletions do not hamper their ability to replicate in the dysregulated tumour environment; however, they prevent replication in adjacent or distant normal tissue.

A yet more exciting development over the last decade has been the incorporation of transgenes into these viruses allowing expression of a variety of exogenous agents in the tumour microenvironment raising the very real prospect of truly immunomodulatory oncolytic viral therapy, and now the discussion has moved onto which transgenes might be the most effective [7].

A range of delivery methods have been employed for these novel agents, chief amongst them has been intratumoural delivery. A broad range of oncolytic viruses have been delivered via intratumoural injection with a measure of success in treating easily reachable solid tumours [8, 9]. However, death from cancer is often the result of inaccessible or metastatic disease. In this context, oncolytic viruses delivered intratumourally rely on viral replication at the tumour site and then systemic dissemination to the distant sites. However, this is transient and often ineffective due to the development of immune responses to the viral infection.

Systemic delivery of oncolytic viruses (OVs) affords the opportunity to treat both the primary tumour and any overt or undiagnosed metastatic deposits simultaneously. As a result, this method of delivery is a very attractive option for the treatment of patients with advanced/metastatic disease or patients with inaccessible disease such as those with pancreatic cancer or brain cancer due to physiological barriers, such as blood-brain barrier.

## 2. Clinical Trials

There have been many clinical trials of a variety of OVs delivered systemically, as summarised in Table 1. Oncolytic adenovirus was one of the first oncolytic viruses to be developed and licensed for treatment of cancer [8, 24]. The first generation of oncolytic adenovirus, ONYX-015 (also known as *dl*1520, H101 in China), is a genetically modified adenovirus with deletion of the 55 kD gene in the E1B region. Nemunaitis et al. [10] in 2001 performed a dose escalation study using this agent in patients with advanced carcinoma with lung metastases. They demonstrated that ONYX-015 was safe to deliver systemically with no toxicity up to doses of 2 × 10^13^ particles, but the study was not designed for objective tumour responses. Also commencing in 2001, a succession of studies delivered ONYX-015 via hepatic artery infusion for the treatment of metastatic colorectal carcinoma with liver deposits [11–13]. In the first of these trials, a phase I dose escalation study, one patient (9%) responded after combination therapy with conventional chemotherapy and two patients (18%) had stable disease lasting several months [11]. In a larger phase II follow-up trial, three patients (11%) had partial responses, nine (33%) had stable disease, and eleven (41%) patients had progressive disease [12]. A final phase II trial by this group demonstrated similar results to the previous studies with overall median survival of 10.7 months with two patients (8%) having a partial response and a further eleven (46%) having stable disease [13]. Of those with stable disease the median survival was prolonged to nineteen months. In a different study, Small et al. [14] treated patients with hormone-refractory metastatic prostate cancer using a single intravenous infusion. Unlike ONYX-015, the adenovirus (CG7870) in this trial was modified so that E1A was under the control of the rat probasin promoter and E1B was under the control of the PSA promoter-enhancer, thus making it prostate specific. Results from this trial were disappointing with no complete nor partial responses, although five patients (22%) did have a 25% to 49% reduction in their serum PSA values.

PV701 is a naturally attenuated Newcastle disease virus, which has been used systemically in a number of clinical trials between 2002 and 2007 [15–18]. Three of these trials were phase I studies in patients with a variety of advanced/metastatic solid tumours [15, 16, 18]. In the Pecora et al. [15] study in 2002, 62 patients were assessed for a tumour response and two patients (3%) had a major response and 14 patients (23%) had stable disease for 4–30 months. Hotte et al. [18] performed a small phase I study and although not designed to assess efficacy, four major (22%) and two minor (11%) responses to the treatment were observed. A similarly sized trial by Laurie et al. [16] in 2006 reported stable disease in four patients (25%) for greater than six months. Freeman et al. [17] investigated the safety of using Newcastle disease virus in patients with recurrent glioblastoma multiforme and as with the other studies the treatment was well tolerated but the efficacy was again disappointing with only one patient (7%) having a complete response.

NV1020 is a Herpes Simplex virus type 1 with deletions of the latency factors ICPO and ICP4, and only one copy of its virulence factor y134.5. Another element of safety is the insertion of the *α*4 promoter to control the HSV-1 TK gene expression, which sensitises the virus to antiviral drugs such as acyclovir. One phase I trial [19, 20] delivering NV1020 via hepatic artery infusion in patients with hepatic metastases from colorectal primaries refractory to first-line treatment reported seven patients (58%) with stable disease and two patients showing a partial response. Median survival in this group was 25 months. Another trial by Geevarghese et al. [21] in 2010 again delivered NV1020 by hepatic artery infusion in patients with advanced metastatic colorectal carcinoma but this time followed by conventional chemotherapy. After completion of the combined approach, there was a 68% response rate, with one patient with a partial response and fourteen patients with stable disease. Median survival in this study was 11.8 months.

Interrogation of the various clinical trial registration sites (http://www.clinicaltrials.gov/, WHO trials register, https://www.clinicaltrialsregister.eu/, http://www.controlled-trials.com/) reveals that there are no ongoing nor pending trials systemically delivering Adenovirus, Newcastle disease virus, or Herpes Simplex virus type 1. 

Reolysin is a type 3 Dearing Reovirus in its wild-type form. Vidal et al. [23] completed the only trial using systemic delivery in 2008. They performed a phase I dose escalation study assessing the safety of a variety of doses. As such they observed no dose-limiting toxicity, and they further comment that antitumour activity was observed both radiologically and by tumour markers. However, no objective radiologic responses were observed in terms of Response Evaluation Criteria in Solid Tumours. Despite this they did report that eight patients showed disease stabilisation. There are also two phase II trials and one phase III trial that have been registered (NCT01166542, NCT01199263, NCT01280058). The phase III trial is in patients with metastatic or recurrent squamous cell carcinoma of the head and neck, whereas the two phase II trials are in recurrent ovarian/fallopian tube cancer and recurrent pancreatic cancer, respectively. All these trials are still recruiting (Table 2).

JX-594 is a Vaccinia virus based on the Wyeth strain with a thymidine kinase (TK) deletion and the insertion of human granulocyte macrophage colony stimulating factor (hGM-CSF) gene and Lac-Z into the TK-deleted region. These transgenes are under the control of pE/L and p7.5 promoters, respectively. Jennerex Biotherapeutics Inc. has reported the results of one trial using this agent systemically in patients with unresectable primary hepatocellular carcinoma. They performed a phase I safety study delivering JX-594 initially systemically then intratumourally with subsequent sorafenib treatment. Seven out of nine of their patients were suitable to be assessed: in six patients (67%), the tumours necrosed, and of these five patients (56%) had stable disease and one patient (11%) had a partial response. They have recently reported the results of another dose escalation study using JX-594 in patients with metastatic solid tumour disease, which was refractory to conventional therapy. The treatment was well tolerated and at higher doses of virus (1 × 10^7^ to 3 × 10^7^ PFU/kg), and they demonstrated that JX-594 can selectively infect, replicate, and express transgene products in target tumour tissue whilst sparing normal tissue. Although the study was not designed for efficacy, one patient had partial response [22]. Jennerex Biotherapeutics Inc. has two trials pending with respect to this agent, the details of which are illustrated in Table 2.

In general these clinical trials have shown that oncolytic viruses can be delivered systemically with limited toxicity and latency. However, what they have not shown, and indeed were not powered to show, is that these agents are efficacious at treating either the primary tumour or metastatic disease. There is a complete lack of appropriately powered phase IIb or phase III trials using OVs delivered systemically, although there are a few pending for Reovirus. The data that are available demonstrate that systemically delivered oncolytic viruses offer only modest improvements, if at all, over and above conventional second-line therapy. Clearly if intravenously delivered OVs are to play a part in the future treatment of advanced cancer, there needs to be dramatic improvement.

## 3. Barriers to Systemic Delivery of Oncolytic Viruses

**Figure 1:**
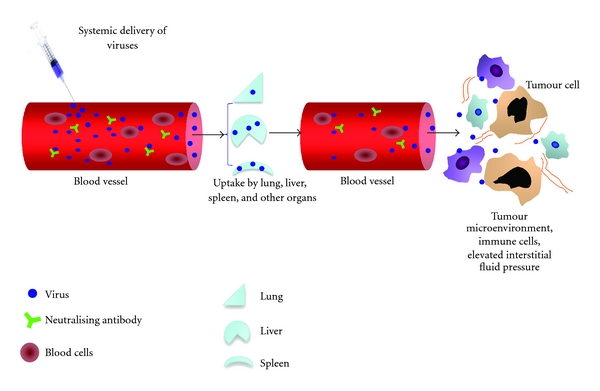
Hurdles of systemic delivery of oncolytic viruses to tumour cells. After intravenous injection, viruses are neutralised by pre-existing antibodies and complement activation. Oncolytic viruses also interact with blood cells. Sequestration into other organs and the reticuloendothelial system is a particular problem, often with resulting toxicities. Macrophages in the lung, liver (aka kupffer cells), and spleen are major players to clear oncolytic viruses after systemic delivery. From the blood stream, viruses have to pass through a mixture of extracellular matrix and cells (including normal and immune cells) before reaching the tumour. The connective tissue of the tumour matrix is important in the regulation and creation of the tumour vasculature; the tumour vasculature itself and interstitial pressures are also key factors involved in the ability of the virus to penetrate the tumour mass.

There are many obstacles to successful systemic delivery of viruses; host defences limit most oncolytic viruses' ability to infect tumours after systemic administration. Blood cells, complement, antibodies, and antiviral cytokines [25], as well as nonspecific uptake by other tissues such as the lung, liver and spleen, tissue-resident macrophages, and additionally poor virus escape from the vascular compartment [3] are the main barriers to systemic delivery of oncolytic viruses (Figure 1). Clearly, in order for this method to be effective, the virus must persist in the circulation without depletion or degradation while selectively infecting tumour cells.

## 4. Neutralising Antibodies

Preexisting immunity is a major problem for systemically delivered viruses whether this has developed due to the ubiquitous nature of the virus, previous immunization, or prior oncolytic viral therapy. Vaccinia virus was used in the worldwide immunisation program for the eradication of smallpox and so many people who are now developing cancer have a preexisting immunity to this OV. Reovirus is universally present within the environment and as a result many people have immunity to it [26, 27]. Furthermore, White et al. [28] have demonstrated that the antibody titre to Reovirus increases dramatically after systemic delivery and others have shown that the presence of these antibodies significantly impairs effective intravenous administration [29, 30]. One simple strategy for overcoming this problem has been to sequentially deliver related viruses with different serotypes or chimeric viruses [31].

Nature has already provided several solutions when considering the significant hurdles to effective systemic delivery with regards to Vaccinia virus, which can potentially be delivered systemically [32] since the Extracellular Enveloped Virus (EEV) form shrouds itself in a host cell-derived envelope and thus can evade both complement and neutralising antibodies [33–36]. Indeed, strains of Vaccinia virus can be engineered that produce more of this immune-evasive form [37]. However, in the clinical setting, it is the intracellular mature virion (IMV) form of the virus that will potentially be injected systemically, and it is this form that must successfully reach the target tissue before any EEV form can be produced. IMV—unlike EEV—is highly immunogenic and is rapidly cleared from the organism if intravenously delivered.

Clearly methods need to be developed that can overcome this acquired immunity. One such strategy is the so-called “Trojan Horse” technique, where cells are taken from the model organism infected with the OV *ex vivo* and then reinfused. Yotnda et al. created transgenic cytotoxic T lymphocytes (CTL), which were transduced with the adenoviral E1 gene under the control of the cell activation-dependent CD40 ligand promoter. The CTLs were transduced *ex vivo* with a conditionally replicating chimera of Adenovirus 5 with the fiber protein of Ad35. This was added as the Ad35 fiber protein can infect cells through a coxsackie and adenovirus receptor-(CAR-) independent method this is required as there is low expression of CAR on CTLs. The transgenic CTL was specifically targeted, and upon binding and subsequent activation, Adenovirus was produced. This occurred since upon activation of the CTL by its specific antigen, the AKNA transcription factor is transiently expressed driving CD40 and E1A expression. Thus by this mechanism, Adenovirus production is tightly linked to CTL activation by its specific tumour-associated antigen resulting in a tumour-specific delivery of Adenovirus [38]. Work by Ilett et al. [39, 40] has shown that dendritic cells loaded *in vitro* with Reovirus will “deliver” the virus successfully to melanoma cells in the presence of neutralising serum. Furthermore, they have shown that Reoviruses loaded into mature dendritic cells are able to infect tumour sites effectively *in vivo* despite preexisting viral immunity. Other cells have been used as potential viral carriers in preclinical models such as cytokine-induced killer (CIK) cells [41], monocytes [42], endothelial cells [42], mesenchymal stem cells [43–45], T-cells [40, 46, 47], dendritic cells [40], and tumour cells [48–50]. Also, stimulated peripheral blood cells, infected with oncolytic Measles virus, have successfully infected Raji lymphomas or hepatocellular carcinoma in the presence of neutralizing antibodies [42]. However, the “Trojan Horse” strategy may not be effective for brain tumours, for which some carrier cells are not able to pass physiological barriers, such as the blood-brain.

Another interesting approach has been developed by Yotnda et al. [51] in which they encapsulated a conditionally replicating competent plasmid based on ONYX-015 in a liposome. They showed that despite circulating Adenovirus antibodies, the liposome-coated viruses were able to infect subcutaneous tumours in mice.

Fontanellas et al. [52] have attempted to overcome the host immunity which develops after repeated administration of Adenovirus by inhibition of T cells and depletion of B-cells with anti-CD20 antibody. Although this study was not targeted at cancer therapy, they demonstrated that this immunosuppressive regime was successful in facilitating gene transfer to hepatocytes despite preexisting Adenoviral immunity.

Another immunosuppressive strategy is to use cyclophosphamide to modulate antiviral immunity in combination with intravenous Reovirus. This has been evaluated in a preclinical murine model by Qiao et al. [53], in which they reported delivery of 1 × 10^7^ plaque-forming units per milligram of tumour with this regime with only mild toxicity to the mice, whereas without cyclophosphamide, effective seeding of the tumour was not achieved. For this particular regime, cyclophosphamide is often used at a lower dose and would not result in significant side effects while it is combined with oncolytic viruses.

## 5. Complement Activation

Complement activation is an important antiviral mechanism. Vaccinia virus in its EEV form incorporates host proteins within its membrane that may well prevent complement activation [36]. Furthermore, it has long been established that Vaccinia virus secretes a variety of immune-modulating molecules. One of the major secreted proteins is Vaccinia complement control protein (VCP), which binds and inactivates C4b and C3b [54–56] thus inhibiting the classic and alternative pathways of complement activation. Furthermore, there is compelling evidence from a variety of viral infection models that complement activation induces various elements within the adaptive immune system [57–62]. Recent work has suggested that VCP dampens viral antibody responses and reduces the accumulation of CD4+ and CD8+ cells at the site of infection in a complement-dependent manner [63]. This has led to at least one group using VCP to perturb complement activation outside the context of a Vaccinia infection [64] and raises the possibility of using it in combination with other OVs to block complement activation.

Herpes simplex virus type 1 has also evolved strategies to prevent complement activation. HSV-1 secretes glycoprotein E that acts as an IgG Fc receptor and effectively blocks both IgG Fc-mediated complement activation and antibody-dependent cellular cytotoxicity [65]. Also HSV-1 produces glycoprotein C that binds C3b and is also critical in preventing C5 activation [66].

Adenovirus activates the complement system by various mechanisms but recent *in vivo* pre-clinical data suggests that this activation can be effectively reduced by shielding Adenovirus with polyethylene glycol [67]. Another approach to ameliorate complement activation, undertaken in Adenovirus, is to make the virus express soluble CD59 [68] and thus prevent deposition of the membrane attack complex.

## 6. Antiviral Cytokines

Viral infections stimulate a variety of cytokines to be produced (for review see Randall 2008 [69]). These include type 1 interferons (IFN), type 2 IFN, and type 3 IFN [70, 71]. Although these molecules have pleiotropic functions, the main effects are to promote apoptosis in virus-infected cells and induce cellular resistance to viral infection in noninfected cells [72]. Additionally, they recruit elements of the adaptive immune system, such as dendritic cells, leading to potentially lasting immunity [73]. Most oncolytic viruses express proteins that block these IFNs [74–76], or their downstream targets, but the anti-viral response is often still sufficient to prevent intra-tumoral spread of the OV.

As has been mentioned earlier, the “Trojan horse” strategy is a potentially powerful technique for delivering oncolytic viruses systemically. Ahmed et al. demonstrated that mesenchymal stem cells infected *ex vivo *with Adenovirus and then subsequently reinfused had great advantages in terms of delivery particularly with respect to attenuating the IFN-gamma response at the tumour site since mesenchymal stem cells suppress activated T-cells [77]. Another strategy to overcome the antiviral cytokines is to pretreat with histone deacetylase inhibitors, which induce epigenetic changes that blunt antiviral cytokine responses at the tumour sites and have been shown to greatly improve the effectiveness of OV therapy [78, 79].

## 7. Nonspecific Uptake by Other Tissues Such as the Liver and Spleen

It is known that many viruses are either filtered or taken up by the lung, liver, or spleen thus reducing systemic availability. Our group has demonstrated that the spleen is pivotal in the early clearance of systemically delivered Vaccinia virus (unpublished data by James Tysome et al.). Furthermore, up to 90% of Adenovirus type 5 is sequestered from the blood by Kupffer cells [80] and as a result this acts as a major obstacle for the systemic delivery of Adenovirus.

With respect to Adenovirus, several lines of investigation have developed strategies for improving its systemic availability. Shashkova et al. [25] demonstrated in a pre-clinical model that pretreatment with warfarin followed by multiple doses of replication-defective Adenovirus successfully depleted Kupffer cells and prevented hepatocyte binding, thus improving the antitumour efficacy of a subsequent single dose of oncolytic Adenovirus. Another important factor involved in liver sequestration of Adenovirus 5 is the binding of its hexon with blood coagulation factor X. Zhang et al. [31] have developed a hexon-chimeric oncolytic Adenovirus type 5 that has Adenovirus type 48's hexon, which only weakly binds factor X. They have demonstrated that this chimera has a significantly reduced liver uptake.

## 8. Suboptimal Viral Escape from the Vascular Compartment

Adenovirus is known to bind to human erythrocytes [81, 82], and this reduces its therapeutic availability when delivered systemically. Furthermore, it is well known that the neovasculature within solid tumours is very chaotic and abnormally leaky with often markedly raised interstitial pressures leading to reduced viral penetration of the tumour mass. Oncolytic viruses are known to stabilize tumour vasculature directly improving tumour penetrance [83]. Interestingly, other work has shown that the addition of antiangiogenic agents with oncolytic viruses can further normalise the vasculature and improve viral delivery in preclinical models [84, 85]. There is also emerging evidence that blockade of the Hedgehog signaling pathway can affect tumour vasculature [86]. Thus a Hedgehog antagonist may prove to be an effective treatment in combination with a systemically delivered oncolytic virus or indeed incorporated within one as a transgene. Another potential agent that could be incorporated into an OV as a transgene is histidine-rich glycoprotein (HRG) particularly in the context of repeated systemic administrations of OV. This protein has been shown to normalise tumour vasculature through its ability to polarize macrophages from M2-like TAM phenotype to M1-like tumour inhibitory phenotype [87].

## 9. Other Physical Methods to Enhance Systemic Delivery

Microbubbles have been developed as a potential method for enhancing the systemic delivery of a variety of agents including oncolytic viruses. They were first developed to help deliver small molecules to target tissues [88–91]. Microbubbles are ultrasound contrast agents that contain high-molecular weight gases which are less soluble and do not diffuse easily, and as a result the microbubbles persist in the circulation for a few minutes passing through the microcirculation several times [88]. Ultrasound-targeted destruction of the microbubbles allows focused release of the oncolytic virus at the tumour site, and a secondary effect is transient and localised increased cellular permeability which potentially can improve viral infection of the cancer cells [92]. This technique has been used *in vivo* with Adenovirus successfully delivering the virus to the tumour site in mice [93, 94]. The technique has not yet been used with other oncolytic viruses.

## 10. Tissue-Resident Macrophages

To date, most pre-clinical studies examining systemic delivery of Vaccinia virus have used nude mice bearing xenograft tumours. It is now clear that there is a need to assess systemic delivery in an immune-competent model as host immunity is a major barrier. Indeed, results from our group have demonstrated that while Vaccinia virus can effectively infect tumour cells in nude mice after systemic delivery, infection of tumour cells cannot be achieved at similar levels in the immunocompetent model. Concurrently, work in our group revealed that depletion of macrophages by clodronate liposomes dramatically enhanced Vaccinia virus infection of tumours in immunocompetent mice after systemic delivery (unpublished data by James Tysome et al.). This almost completely restored the antitumour potency to the level seen in nude mice. However, clodronate liposomes nonselectively deplete macrophages and therefore potentially diminish any beneficial activity in the tumour microenvironment unrelated to viral clearance. Consequently, this necessitates a search for a novel, more selective agent that could interfere transiently with macrophage function and thus enhance the systemic delivery of Vaccinia virus.

In general, it should be possible to perturb macrophage function at a variety of stages such as their development, recruitment/migration, or blocking their phagocytic function. Several lines of evidence have highlighted an important role for phosphatidylinositol 3-kinases (PI3K) [95–98] in macrophage phagocytosis. These observations imply that PI3K inhibitors may be potential therapeutic agents for enhancement of systemic delivery of Vaccinia virus, and other OVs, by blocking macrophage uptake/clearance of the viruses. One caveat to this is that therapeutic interference in the PI3K pathway may have to be targeted at individual or groups of PI3K isoforms [99]. It is known that mammals have eight isoforms of PI3K, but the specific isoforms of PI3K involved in macrophage phagocytosis and Vaccinia, or other OV, uptake have yet to be elucidated.

## 11. Conclusions

To date, the systemic delivery of oncolytic viruses has been shown to be safe but not efficacious mainly due to immunological factors that facilitate rapid clearance of these agents. There is a range of novel methods that are being developed at a pre-clinical level to overcome these hurdles which have been reported to be successful *in vivo* mainly in murine models.

However, we need to remember that mouse models are just that—they are models, which offer opportunities to investigate the effect of host factors on systemic delivery of oncolytic virus *in vivo*. The major problem is that the host immune responses to some oncolytic viruses in mice are completely different from those in humans reflecting their mutual genetic divergence 65 million years ago. Most importantly, for some oncolytic viruses such as oncolytic adenovirus, murine models of cancer are suboptimal as murine tissue and cells do not support adenovirus replication. Therefore, the information derived from these models about the host immune response to oncolytic adenovirus is certainly different and nonrepresentative of the situation in humans. Given these limitations, the next step will be the commencement of clinical trials combining these methods with systemically delivered oncolytic viruses, investigating whether these strategies work in humans. Several agents that can enhance the systemic delivery of oncolytic viruses have been separately used or tested in clinical trials. It is conceivable that a combination strategy to enhance the systemic delivery of oncolytic viruses should and will be employed in the near future. This strategy may provide an effective therapeutic approach for treatment of primary tumours, the metastatic deposits, and tumour entities, which are not easily accessible for conventional therapeutic agents because of physiological barriers. The blood-brain barrier is one such obstacle, which it has been demonstrated that several oncolytic viruses have been able to pass identifying them as potential candidates in the treatment of brain tumours.

In conclusion, if an optimal approach to enhance the systemic delivery of oncolytic viruses can be achieved by rationally targeting different factors, the outcome for treatment of advanced cancers would be dramatically improved.

## Figures and Tables

**Table 1:** Completed clinical trials using systemically delivered oncolytic viruses for the treatment of solid tumours.

Viral agent	Virus species	Modifications	Cancer type	Pt no.	Treatment regime	Toxicity	Results	References
ONYX-015	Adenovirus	E1B-55 kD-ve	Advanced carcinoma with lung metastasis	10	Phase I, dose-escalation study	No dose-limiting toxicity was observed. Grade 2-3 toxicities were common (fever, rigors and fatigue).	IV administration was well tolerated up to 2 × 10^13^ particles. Not designed for objective tumour responses.	Nemunaitis et al., 2001 [10]

ONYX-015	Adenovirus	E1B-55 kD-ve	Liver metastases from gastrointestinal carcinoma	11	Phase I, dose-escalation study hepatic artery infusion	No dose-limiting toxicity. Commonly mild to moderate fever, rigors and fatigue.	Objective response with chemotherapy + virus in a patient who was refractory to both 5-FU and ONYX-015 as single agents. 2 high dose patients had stable disease on combination therapy lasting from 7 to 17 months.	Reid et al., 2001 [11]

Onyx-015	Adenovirus	E1B-55 kD-ve	Metastatic liver deposits from gastrointestinal primaries	27	Phase II, hepatic artery infusion in combination with 5 FU and leucovorin	2 patients had reversible grade 3/4 hyperbilirubinemia and 1 patient had a reversible severe systemic inflammatory response.	3 patients with partial responses, 4 with minimal responses, 9 with stable disease, and 11 with progressive disease. Unclear from this study whether observed responses were due to viral treatment or chemotherapy or combination.	Reid et al., 2002 [12]

ONYX-015	Adenovirus	E1B-55 kD-ve	Metastatic colorectal cancer refractory to conventional therapy	24	Phase I/II, hepatic artery infusion	No dose-limiting toxicity was observed.	Overall median survival was 10.7 months. 2 patients had partial responses (tumur vol reductions of 66% and 72%). 11 patients had stable disease after viral treatment and median survival in this group was 19 months.	Reid et al., 2005 [13]

CG7870	Adenovirus	E1A under control of the rat probasin promoter E1B under control of the PSA promoter-enhancer	Hormone-refractory metastatic prostate cancer	23	Phase I, single intravenous infusion	Mostly grade 1 or 2 flulike symptoms were observed. There were 8 grade 3 events (fever or fatigue). At higher doses, asymptomatic grade 1 or 2 transaminitis were reported.	No partial or complete tumour responses were observed. 5 patients had a decrease in serum PSA of 25% to 49% following a single treatment.	Small et al., 2006 [14]

PV701	Newcastle disease virus	Naturally attenuated	Advanced or metastatic solid cancers that were refractory to standard treatment	79	Patients were recruited into 4 different IV dosing regimes.	Grade 3 fever in 11% flu-like symptoms	62 patients assessed for response, 2 major responses, 14 had no disease progression for 4–30 months.	Pecora et al., 2002 [15]

PV701	Newcastle disease virus	Naturally attenuated	Pallitive solid tumors	16	Phase 1	No dose-limiting toxicities. Mild flu-like symptoms were common.	Four patients had stable disease for ≥6 months.	Laurie et al., 2006 [16]

NDV-HUJ	Newcastle disease virus	Naturally attenuated	Recurrent glioblastoma multiforme	14	Phase I/II	5 patients had grade I/II constitutional fever.	One patient achieved a complete response.	Freeman et al., 2006 [17]

PV701	Newcastle disease virus	Naturally attenuated	Advanced chemorefractory cancer	18	Phase 1	Mild or moderate in severity, and self-limiting	Not design for assessment of response but 4 major and 2 minor tumour responses were observed.	Hotte et al., 2007 [18]

NV1020	Herpes simplex virus type 1	ICP0 & ICP4-ve Only 1 copy of *γ*134.5 Transgene inserted HSV-1 TK gene (*α*4)	Hepatic colorectal metastases refractory to first-line chemotherapy	12	Phase I, dose-escalation study, hepatic artery infusion	Mild or moderate in severity, and self-limiting	Tumour response assessed at 28 days after viral delivery. 7 patients had stable disease. 2 patients had a partial response (tumour vol reductions of 39% and 20%). Median survival for this group was 25 months.	Kemeny et al., 2006 [19] Fong et al., 2009 [20]

NV1020	Herpes simplex virus type 1	ICP0 & ICP4-ve Only 1 copy of *γ*134.5 Transgene inserted HSV-1 TK gene (*α*4)	Advanced metastatic colorectal	Phase 1 = 13, Phase 2 = 19	Phase I/II study, hepatic artery infusion treatment followed by two or more cycles of conventional chemotherapy	Mild-to-moderate febrile reactions after each NV1020 infusion. Grade 3/4 transient lymphopenia in two patients	After completion of NV1020 administration, 50% showed stable disease. Best overall tumor control rate after completion of combined therapy was 68% (1 partial response, 14 stable disease). Median time to progression was 6.4 months. Median overall survival was 11.8 months. One-year survival was 47.2%.	Geevarghese et al., 2010 [21]

JX-594	Vaccinia—Wyeth	TK-ve Transgene inserted in TK region hGM-CSF (pE/L) and Lac-Z (p7.5)	Unresectable primary hepatocellular carcinoma	9	Phase 2, pilot safety study IV then IT injection followed by sorafenib treatment	They assert viral treatment was well tolerated and sorafenib side effects were consistent with previously reported.	7 out of 9 were able to be assessed for response. Of these, 6 achieved necrotic responses. 5 had stable disease and 1 had a partial response.	http://www.clinicaltrials.gov/ Jennerex Biotherapeutics, Inc. NCT01171651 http://www.jennerex.com/pr_091310.html

JX-594	Vaccinia—Wyeth	TK-ve Transgene inserted in TK region hGM-CSF (pE/L) and Lac-Z (p7.5)	Metastatic solid tumour disease which is refractory to conventional therapy specifically: Melanoma, Lung Cancer, Renal cell cancer, SCC of head and neck	23	Phase I, Dose Escalation Study	No dose-limiting toxicities. Mild flu-like symptoms were common.	Demonstrated that at the higher doses used (1 × 10^7^ to 3 × 10^7^ PFU/kg) JX-594 can selectively infect, replicate and express transgene products in target tumour tissue whilst sparing normal tissue. Although not designed for efficacy, one patient had partial response.	http://www.clinicaltrials.gov/ Jennerex Biotherapeutics, Inc. NCT00625456 Breitbach et al., 2011 [22]

Reolysin	Reovirus—type 3 Dearing	Wild-type	Advanced or metastatic solid cancers that were refractory to standard treatment	33	Phase 1, Dose Escalation study	No dose-limiting toxicity was observed. Grade 1-2 toxicities were common (fever, fatigue and headache).	Antitumor activity was observed radiologically and by tumor markers.	Vidal et al., 2008 [23]

**Table 2:** Ongoing or pending clinical trials using systemically delivered oncolytic viruses for the treatment of solid tumours.

Viral agent	Virus species	Modifications	Cancer type	Patient No.	Treatment regime	Status	References
MV-NIS	oncolytic measles virus—Edmonston vaccine strain	Transgene: thyroidal sodium iodide symporter	Recurrent or refractory multiple myeloma	Est 54 Enrolment closes June 2012	Phase I trial Safe and dose escalation study of vaccine therapy when given with or without cyclophosphamide	Recruiting	http://www.clinicaltrials.gov/ Mayo Clinic NCT00450814

JX-594	Vaccinia—Wyeth	TK-ve Transgene inserted in TK region hGM-CSF (pE/L) and Lac-Z (p7.5)	Metastatic colorectal carcinoma	Est 20 Not open for enrolment yet	Neoadjuvant Phase 2a trial IV or IT injection followed by surgical resection of metastatic hepatic deposits	Not yet open	http://www.clinicaltrials.gov/ Jennerex Biotherapeutics, Inc. NCT01329809

JX-594	Vaccinia—Wyeth	TK-ve Transgene inserted in TK region hGM-CSF (pE/L) and Lac-Z (p7.5)	Metastatic colorectal carcinoma refractory to or intolerant of oxaliplatin, irinotecan, and Erbitux	Est 15 Enrolment closes June 2012	Phase 1b dose escalation study IV injection biweekly (to evaluate the safety and tolerability of JX-594)	Results pending	http://www.clinicaltrials.gov/ Jennerex Biotherapeutics, Inc. NCT01380600

Reolysin	Reovirus—type 3 Dearing	Wild-type	Metastatic or recurrent SCC of H&N	Est 280 enrolment closes Dec 2012	Phase 3 IV administration in combination with paclitaxel and carboplatin versus chemotherapy treatment alone	Recruiting	http://www.clinicaltrials.gov/ Oncolytics Biotech NCT01166542

Reolysin	Reovirus—type 3 Dearing	Wild-type	Recurrent or persistent ovarian, fallopian Tube, or primary peritoneal cancer	Est 110 enrolment closes December 2012	Phase II weekly paclitaxel versus weekly paclitaxel with IV Reovirus	Recruiting	http://www.clinicaltrials.gov/ Gynecologic Oncology GroupNCT01199263

Reolysin	Reovirus—type 3 Dearing	Wild-type	Recurrent or metastatic pancreatic cancer	Est 70 enrolment closes December 2013	Phase II carboplatin, paclitaxel plus Reovirus versus carboplatin and paclitaxel	Recruiting	http://www.clinicaltrials.gov/ Arthur G. James Cancer Hospital & Richard J. Solove Research Institute NCT01280058
